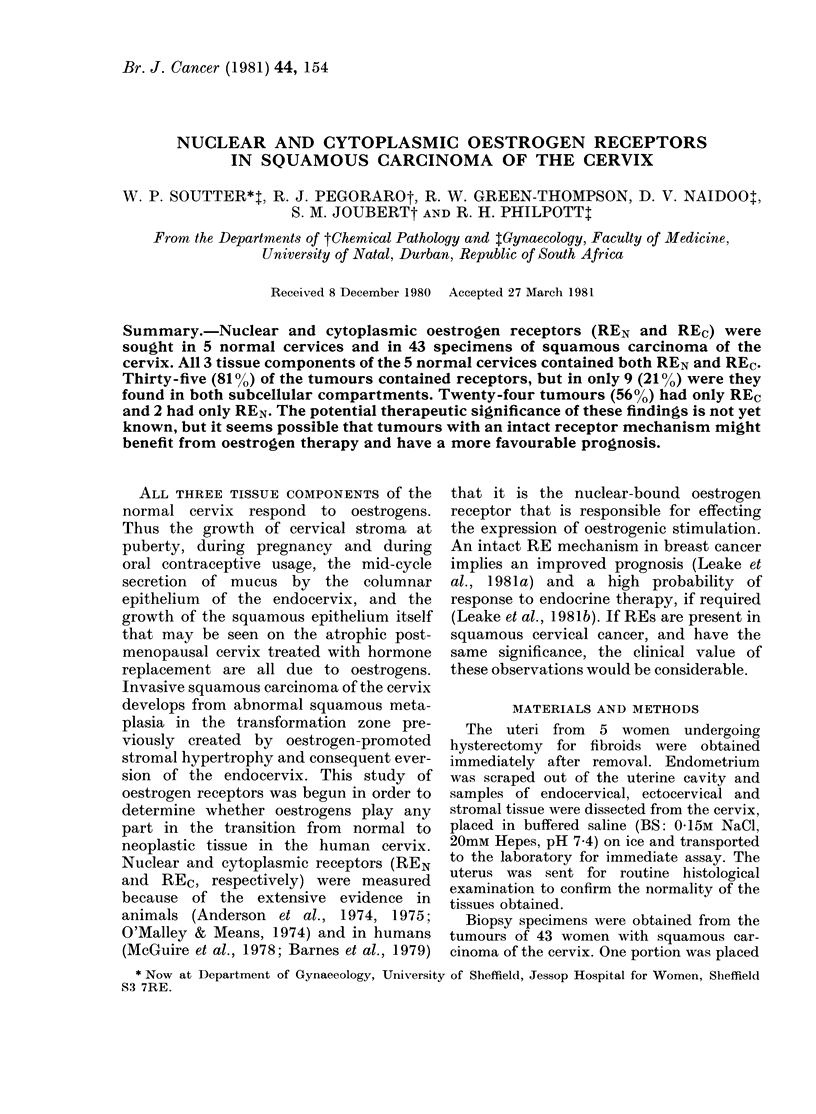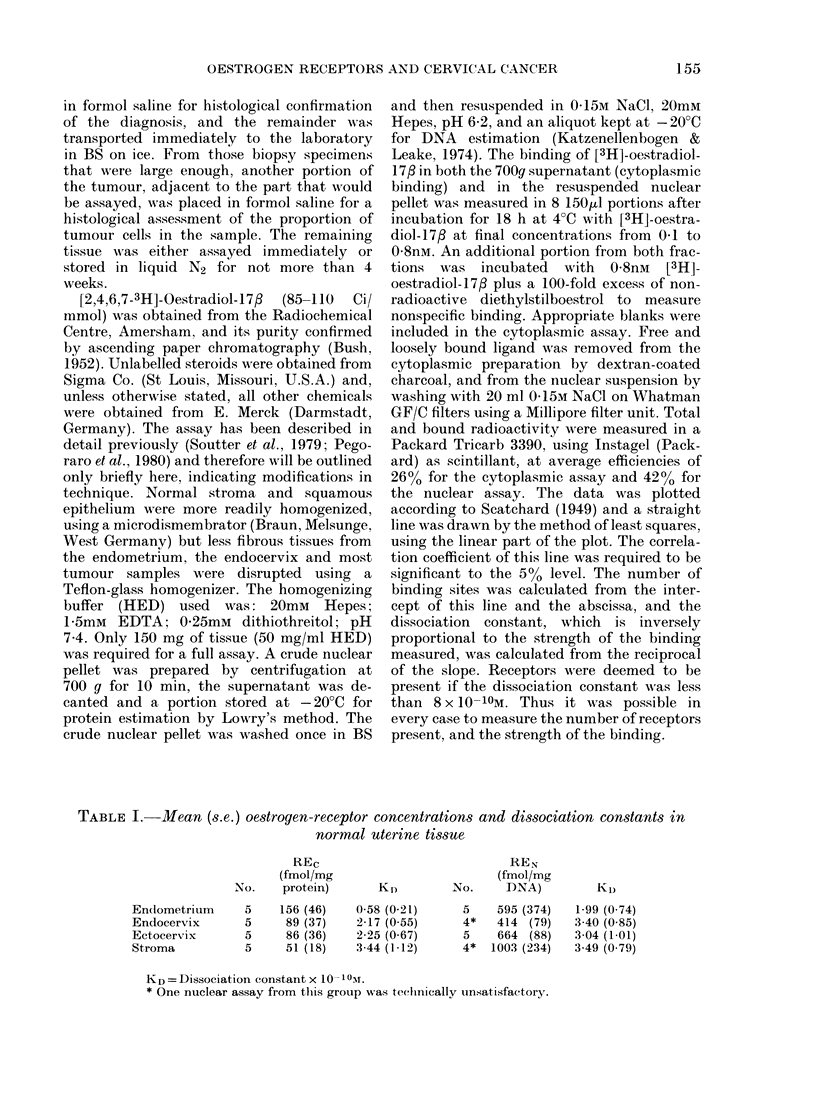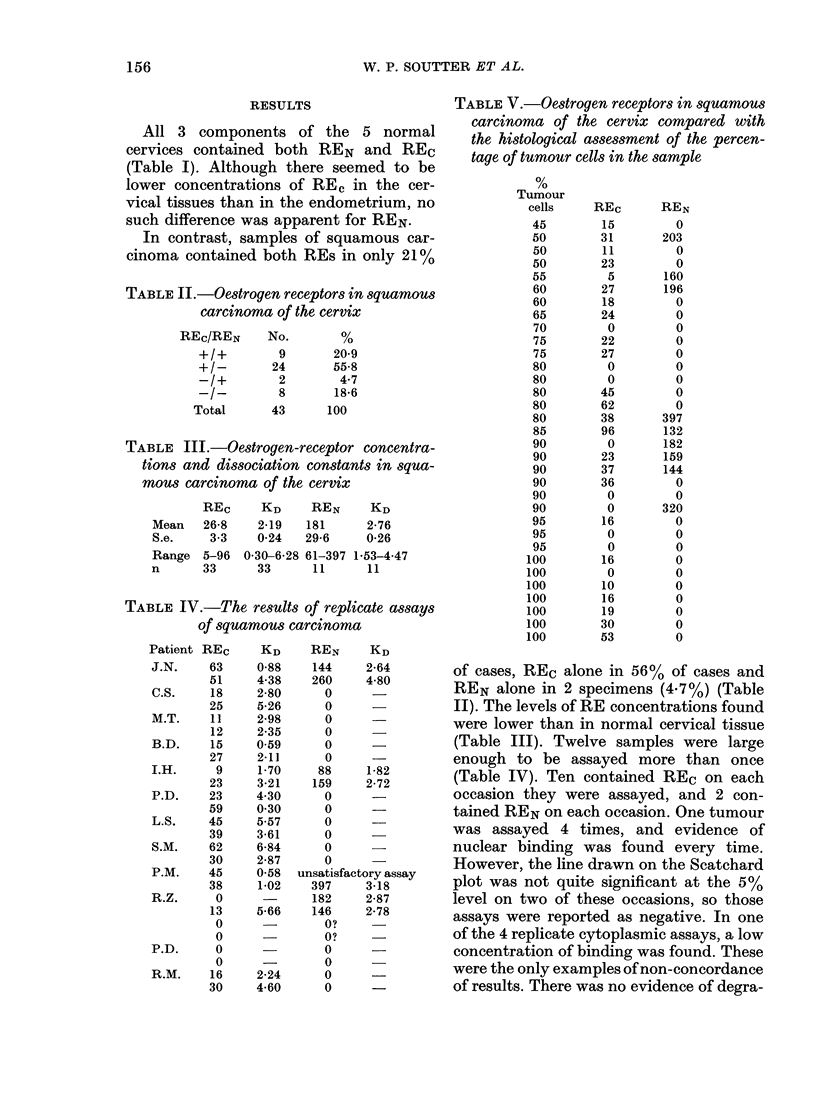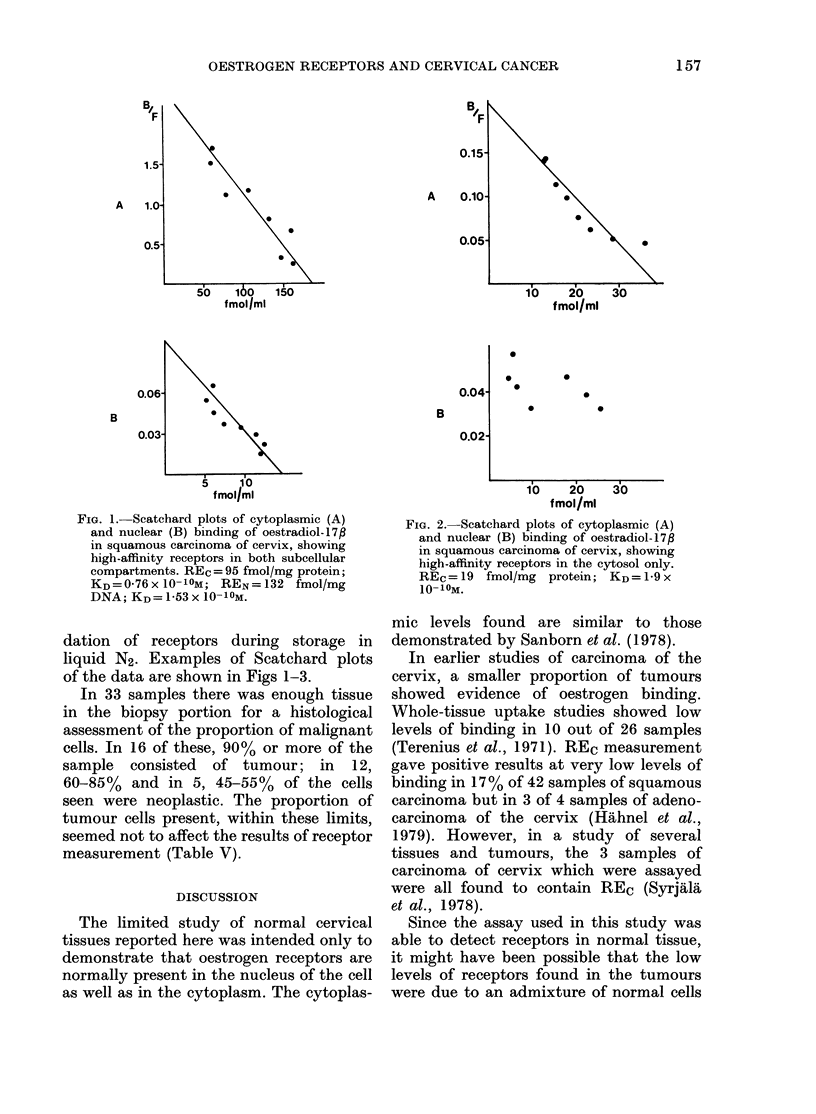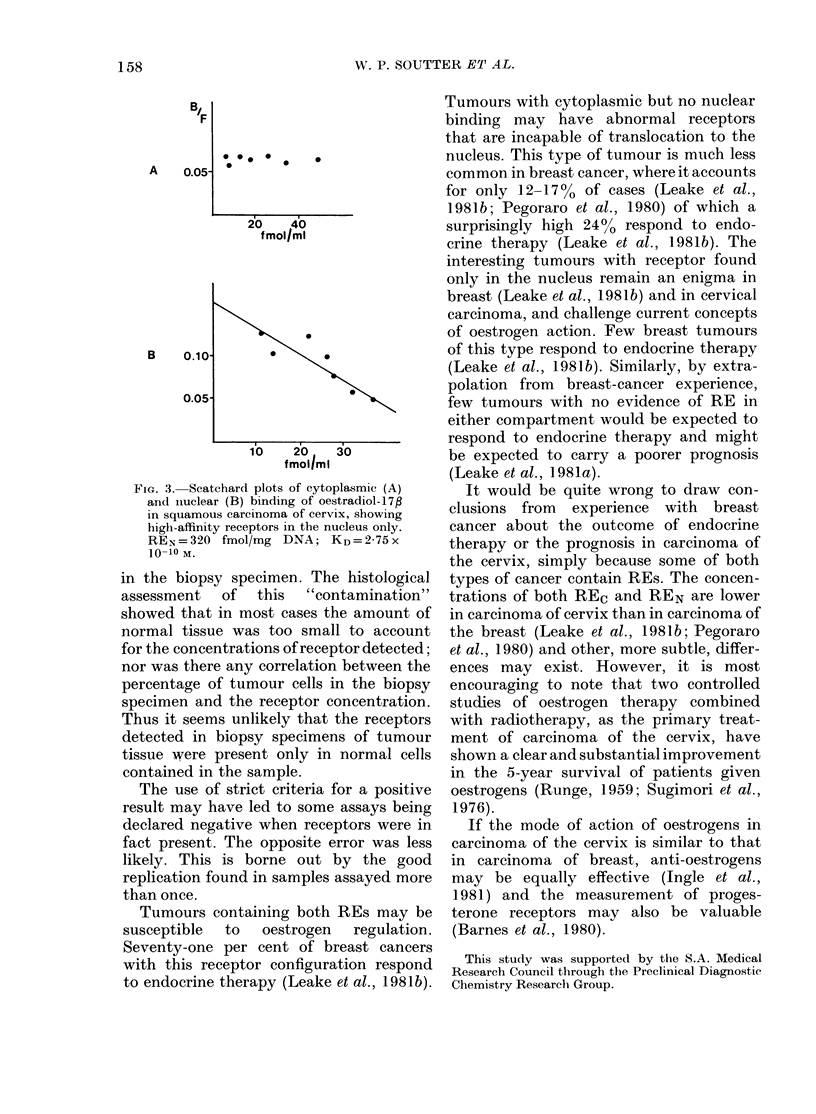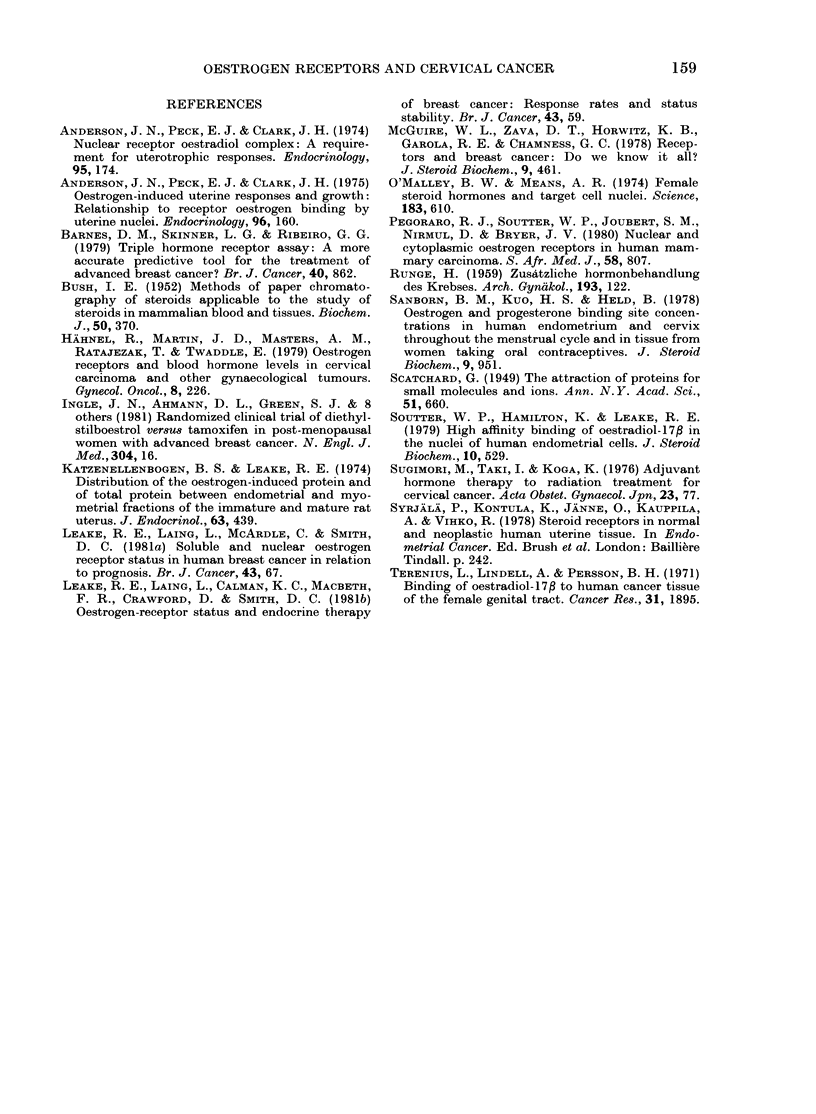# Nuclear and cytoplasmic oestrogen receptors in squamous carcinoma of the cervix.

**DOI:** 10.1038/bjc.1981.165

**Published:** 1981-08

**Authors:** W. P. Soutter, R. J. Pegoraro, R. W. Green-Thompson, D. V. Naidoo, S. M. Joubert, R. H. Philpott

## Abstract

Nuclear and cytoplasmic oestrogen receptors (REN and REC) were sought in 5 normal cervices and in 43 specimens of squamous carcinoma of the cervix. All 3 tissues components of the 5 normal cervices contained both REN and REC. Thirty-five (81%) of the tumours contained receptors, but in only 9 (21%) were they found in both subcellular compartments. Twenty-four tumours (56%) had only REC and 2 had only REN. The potential therapeutic significance of these findings is not yet known, but it seems possible that tumours with an intact receptor mechanism might benefit from oestrogen therapy and have a more favourable prognosis.


*Br. J. Cancer* (1981) **44**, 154

### NUCLEAR AND CYTOPLASMIC OESTROGEN RECEPTORS IN SQUAMOUS CARCINOMA OF THE CERVIX

W. P. SOUTTER*‡, R. J. PEGORARO†, R. W. GREEN-THOMPSON, D. V. NAIDOO‡, S. M. JOUBERT† AND R. H. PHILPOTT‡

*From the Departments of †Chemical Pathology and ‡Gynaecology, Faculty of Medicine, University of Natal, Durban, Republic of South Africa*

Received 8 December 1980 Accepted 27 March 1981

**Summary.—Nuclear and cytoplasmic oestrogen receptors ($RE_N$ and $RE_C$) were sought in 5 normal cervices and in 43 specimens of squamous carcinoma of the cervix. All 3 tissue components of the 5 normal cervices contained both $RE_N$ and $RE_C$. Thirty-five (81%) of the tumours contained receptors, but in only 9 (21%) were they found in both subcellular compartments. Twenty-four tumours (56%) had only $RE_C$ and 2 had only $RE_N$. The potential therapeutic significance of these findings is not yet known, but it seems possible that tumours with an intact receptor mechanism might benefit from oestrogen therapy and have a more favourable prognosis.**

ALL THREE TISSUE COMPONENTS of the normal cervix respond to oestrogens. Thus the growth of cervical stroma at puberty, during pregnancy and during oral contraceptive usage, the mid-cycle secretion of mucus by the columnar epithelium of the endocervix, and the growth of the squamous epithelium itself that may be seen on the atrophic post-menopausal cervix treated with hormone replacement are all due to oestrogens. Invasive squamous carcinoma of the cervix develops from abnormal squamous metaplasia in the transformation zone previously created by oestrogen-promoted stromal hypertrophy and consequent eversion of the endocervix. This study of oestrogen receptors was begun in order to determine whether oestrogens play any part in the transition from normal to neoplastic tissue in the human cervix. Nuclear and cytoplasmic receptors ($RE_N$ and $RE_C$, respectively) were measured because of the extensive evidence in animals (Anderson *et al.*, 1974, 1975; O'Malley & Means, 1974) and in humans (McGuire *et al.*, 1978; Barnes *et al.*, 1979) that it is the nuclear-bound oestrogen receptor that is responsible for effecting the expression of oestrogenic stimulation. An intact RE mechanism in breast cancer implies an improved prognosis (Leake *et al.*, 1981*a*) and a high probability of response to endocrine therapy, if required (Leake *et al.*, 1981*b*). If REs are present in squamous cervical cancer, and have the same significance, the clinical value of these observations would be considerable.

### MATERIALS AND METHODS

The uteri from 5 women undergoing hysterectomy for fibroids were obtained immediately after removal. Endometrium was scraped out of the uterine cavity and samples of endocervical, ectocervical and stromal tissue were dissected from the cervix, placed in buffered saline (BS: 0·15M NaCl, 20mM Hepes, pH 7·4) on ice and transported to the laboratory for immediate assay. The uterus was sent for routine histological examination to confirm the normality of the tissues obtained.

Biopsy specimens were obtained from the tumours of 43 women with squamous carcinoma of the cervix. One portion was placed

* Now at Department of Gynaecology, University of Sheffield, Jessop Hospital for Women, Sheffield S3 7RE.

in formol saline for histological confirmation of the diagnosis, and the remainder was transported immediately to the laboratory in BS on ice. From those biopsy specimens that were large enough, another portion of the tumour, adjacent to the part that would be assayed, was placed in formol saline for a histological assessment of the proportion of tumour cells in the sample. The remaining tissue was either assayed immediately or stored in liquid $N_2$ for not more than 4 weeks.

[2,4,6,7-$^3$H]-Oestradiol-17$\beta$ (85–110 Ci/mmol) was obtained from the Radiochemical Centre, Amersham, and its purity confirmed by ascending paper chromatography (Bush, 1952). Unlabelled steroids were obtained from Sigma Co. (St Louis, Missouri, U.S.A.) and, unless otherwise stated, all other chemicals were obtained from E. Merck (Darmstadt, Germany). The assay has been described in detail previously (Soutter *et al.*, 1979; Pegoraro *et al.*, 1980) and therefore will be outlined only briefly here, indicating modifications in technique. Normal stroma and squamous epithelium were more readily homogenized, using a microdismembrator (Braun, Melsunge, West Germany) but less fibrous tissues from the endometrium, the endocervix and most tumour samples were disrupted using a Teflon-glass homogenizer. The homogenizing buffer (HED) used was: 20mM Hepes; 1·5mM EDTA; 0·25mM dithiothreitol; pH 7·4. Only 150 mg of tissue (50 mg/ml HED) was required for a full assay. A crude nuclear pellet was prepared by centrifugation at 700 *g* for 10 min, the supernatant was decanted and a portion stored at −20°C for protein estimation by Lowry's method. The crude nuclear pellet was washed once in BS and then resuspended in 0·15M NaCl, 20mM Hepes, pH 6·2, and an aliquot kept at −20°C for DNA estimation (Katzenellenbogen & Leake, 1974). The binding of [$^3$H]-oestradiol-17$\beta$ in both the 700*g* supernatant (cytoplasmic binding) and in the resuspended nuclear pellet was measured in 8 150$\mu$l portions after incubation for 18 h at 4°C with [$^3$H]-oestradiol-17$\beta$ at final concentrations from 0·1 to 0·8nM. An additional portion from both fractions was incubated with 0·8nM [$^3$H]-oestradiol-17$\beta$ plus a 100-fold excess of non-radioactive diethylstilboestrol to measure nonspecific binding. Appropriate blanks were included in the cytoplasmic assay. Free and loosely bound ligand was removed from the cytoplasmic preparation by dextran-coated charcoal, and from the nuclear suspension by washing with 20 ml 0·15M NaCl on Whatman GF/C filters using a Millipore filter unit. Total and bound radioactivity were measured in a Packard Tricarb 3390, using Instagel (Packard) as scintillant, at average efficiencies of 26% for the cytoplasmic assay and 42% for the nuclear assay. The data was plotted according to Scatchard (1949) and a straight line was drawn by the method of least squares, using the linear part of the plot. The correlation coefficient of this line was required to be significant to the 5% level. The number of binding sites was calculated from the intercept of this line and the abscissa, and the dissociation constant, which is inversely proportional to the strength of the binding measured, was calculated from the reciprocal of the slope. Receptors were deemed to be present if the dissociation constant was less than $8 \times 10^{-10}$M. Thus it was possible in every case to measure the number of receptors present, and the strength of the binding.

TABLE I.—*Mean (s.e.) oestrogen-receptor concentrations and dissociation constants in normal uterine tissue*

| | No. | $RE_C$ (fmol/mg protein) | $K_D$ | No. | $RE_N$ (fmol/mg DNA) | $K_D$ |
|---|---|---|---|---|---|---|
| Endometrium | 5 | 156 (46) | 0·58 (0·21) | 5 | 595 (374) | 1·99 (0·74) |
| Endocervix | 5 | 89 (37) | 2·17 (0·55) | 4* | 414 (79) | 3·40 (0·85) |
| Ectocervix | 5 | 86 (36) | 2·25 (0·67) | 5 | 664 (88) | 3·04 (1·01) |
| Stroma | 5 | 51 (18) | 3·44 (1·12) | 4* | 1003 (234) | 3·49 (0·79) |

$K_D$ = Dissociation constant × $10^{-10}$M.

* One nuclear assay from this group was technically unsatisfactory.

### RESULTS

All 3 components of the 5 normal cervices contained both $RE_N$ and $RE_C$ (Table I). Although there seemed to be lower concentrations of $RE_c$ in the cervical tissues than in the endometrium, no such difference was apparent for $RE_N$.

In contrast, samples of squamous carcinoma contained both REs in only 21% of cases, $RE_C$ alone in 56% of cases and $RE_N$ alone in 2 specimens (4·7%) (Table II). The levels of RE concentrations found were lower than in normal cervical tissue (Table III). Twelve samples were large enough to be assayed more than once (Table IV). Ten contained $RE_C$ on each occasion they were assayed, and 2 contained $RE_N$ on each occasion. One tumour was assayed 4 times, and evidence of nuclear binding was found every time. However, the line drawn on the Scatchard plot was not quite significant at the 5% level on two of these occasions, so those assays were reported as negative. In one of the 4 replicate cytoplasmic assays, a low concentration of binding was found. These were the only examples of non-concordance of results. There was no evidence of degra-

TABLE II.—*Oestrogen receptors in squamous carcinoma of the cervix*

| $RE_C/RE_N$ | No. | % |
|---|---|---|
| +/+ | 9 | 20·9 |
| +/− | 24 | 55·8 |
| −/+ | 2 | 4·7 |
| −/− | 8 | 18·6 |
| Total | 43 | 100 |

TABLE III.—*Oestrogen-receptor concentrations and dissociation constants in squamous carcinoma of the cervix*

| | $RE_C$ | $K_D$ | $RE_N$ | $K_D$ |
|---|---|---|---|---|
| Mean | 26·8 | 2·19 | 181 | 2·76 |
| S.e. | 3·3 | 0·24 | 29·6 | 0·26 |
| Range | 5–96 | 0·30–6·28 | 61–397 | 1·53–4·47 |
| n | 33 | 33 | 11 | 11 |

TABLE IV.—*The results of replicate assays of squamous carcinoma*

| Patient | $RE_C$ | $K_D$ | $RE_N$ | $K_D$ |
|---|---|---|---|---|
| J.N. | 63 | 0·88 | 144 | 2·64 |
| | 51 | 4·38 | 260 | 4·80 |
| C.S. | 18 | 2·80 | 0 | — |
| | 25 | 5·26 | 0 | — |
| M.T. | 11 | 2·98 | 0 | — |
| | 12 | 2·35 | 0 | — |
| B.D. | 15 | 0·59 | 0 | — |
| | 27 | 2·11 | 0 | — |
| I.H. | 9 | 1·70 | 88 | 1·82 |
| | 23 | 3·21 | 159 | 2·72 |
| P.D. | 23 | 4·30 | 0 | — |
| | 59 | 0·30 | 0 | — |
| L.S. | 45 | 5·57 | 0 | — |
| | 39 | 3·61 | 0 | — |
| S.M. | 62 | 6·84 | 0 | — |
| | 30 | 2·87 | 0 | — |
| P.M. | 45 | 0·58 | unsatisfactory assay | |
| | 38 | 1·02 | 397 | 3·18 |
| R.Z. | 0 | — | 182 | 2·87 |
| | 13 | 5·66 | 146 | 2·78 |
| | 0 | — | 0? | — |
| | 0 | — | 0? | — |
| P.D. | 0 | — | 0 | — |
| | 0 | — | 0 | — |
| R.M. | 16 | 2·24 | 0 | — |
| | 30 | 4·60 | 0 | — |

TABLE V.—*Oestrogen receptors in squamous carcinoma of the cervix compared with the histological assessment of the percentage of tumour cells in the sample*

| % Tumour cells | $RE_C$ | $RE_N$ |
|---|---|---|
| 45 | 15 | 0 |
| 50 | 31 | 203 |
| 50 | 11 | 0 |
| 50 | 23 | 0 |
| 55 | 5 | 160 |
| 60 | 27 | 196 |
| 60 | 18 | 0 |
| 65 | 24 | 0 |
| 70 | 0 | 0 |
| 75 | 22 | 0 |
| 75 | 27 | 0 |
| 80 | 0 | 0 |
| 80 | 0 | 0 |
| 80 | 45 | 0 |
| 80 | 62 | 0 |
| 80 | 38 | 397 |
| 85 | 96 | 132 |
| 90 | 0 | 182 |
| 90 | 23 | 159 |
| 90 | 37 | 144 |
| 90 | 36 | 0 |
| 90 | 0 | 0 |
| 90 | 0 | 320 |
| 95 | 16 | 0 |
| 95 | 0 | 0 |
| 95 | 0 | 0 |
| 100 | 16 | 0 |
| 100 | 0 | 0 |
| 100 | 10 | 0 |
| 100 | 16 | 0 |
| 100 | 19 | 0 |
| 100 | 30 | 0 |
| 100 | 53 | 0 |

FIG. 1.—Scatchard plots of cytoplasmic (A) and nuclear (B) binding of oestradiol-17$\beta$ in squamous carcinoma of cervix, showing high-affinity receptors in both subcellular compartments. $RE_C = 95$ fmol/mg protein; $K_D = 0{\cdot}76 \times 10^{-10}$M; $RE_N = 132$ fmol/mg DNA; $K_D = 1{\cdot}53 \times 10^{-10}$M.

FIG. 2.—Scatchard plots of cytoplasmic (A) and nuclear (B) binding of oestradiol-17$\beta$ in squamous carcinoma of cervix, showing high-affinity receptors in the cytosol only. $RE_C = 19$ fmol/mg protein; $K_D = 1{\cdot}9 \times 10^{-10}$M.

dation of receptors during storage in liquid $N_2$. Examples of Scatchard plots of the data are shown in Figs 1–3.

In 33 samples there was enough tissue in the biopsy portion for a histological assessment of the proportion of malignant cells. In 16 of these, 90% or more of the sample consisted of tumour; in 12, 60–85% and in 5, 45–55% of the cells seen were neoplastic. The proportion of tumour cells present, within these limits, seemed not to affect the results of receptor measurement (Table V).

### DISCUSSION

The limited study of normal cervical tissues reported here was intended only to demonstrate that oestrogen receptors are normally present in the nucleus of the cell as well as in the cytoplasm. The cytoplasmic levels found are similar to those demonstrated by Sanborn *et al.* (1978).

In earlier studies of carcinoma of the cervix, a smaller proportion of tumours showed evidence of oestrogen binding. Whole-tissue uptake studies showed low levels of binding in 10 out of 26 samples (Terenius *et al.*, 1971). $RE_C$ measurement gave positive results at very low levels of binding in 17% of 42 samples of squamous carcinoma but in 3 of 4 samples of adenocarcinoma of the cervix (Hähnel *et al.*, 1979). However, in a study of several tissues and tumours, the 3 samples of carcinoma of cervix which were assayed were all found to contain $RE_C$ (Syrjälä *et al.*, 1978).

Since the assay used in this study was able to detect receptors in normal tissue, it might have been possible that the low levels of receptors found in the tumours were due to an admixture of normal cells

FIG. 3.—Scatchard plots of cytoplasmic (A) and nuclear (B) binding of oestradiol-17$\beta$ in squamous carcinoma of cervix, showing high-affinity receptors in the nucleus only. $RE_N = 320$ fmol/mg DNA; $K_D = 2{\cdot}75 \times 10^{-10}$ M.

in the biopsy specimen. The histological assessment of this "contamination" showed that in most cases the amount of normal tissue was too small to account for the concentrations of receptor detected; nor was there any correlation between the percentage of tumour cells in the biopsy specimen and the receptor concentration. Thus it seems unlikely that the receptors detected in biopsy specimens of tumour tissue were present only in normal cells contained in the sample.

The use of strict criteria for a positive result may have led to some assays being declared negative when receptors were in fact present. The opposite error was less likely. This is borne out by the good replication found in samples assayed more than once.

Tumours containing both REs may be susceptible to oestrogen regulation. Seventy-one per cent of breast cancers with this receptor configuration respond to endocrine therapy (Leake *et al.*, 1981*b*). Tumours with cytoplasmic but no nuclear binding may have abnormal receptors that are incapable of translocation to the nucleus. This type of tumour is much less common in breast cancer, where it accounts for only 12–17% of cases (Leake *et al.*, 1981*b*; Pegoraro *et al.*, 1980) of which a surprisingly high 24% respond to endocrine therapy (Leake *et al.*, 1981*b*). The interesting tumours with receptor found only in the nucleus remain an enigma in breast (Leake *et al.*, 1981*b*) and in cervical carcinoma, and challenge current concepts of oestrogen action. Few breast tumours of this type respond to endocrine therapy (Leake *et al.*, 1981*b*). Similarly, by extrapolation from breast-cancer experience, few tumours with no evidence of RE in either compartment would be expected to respond to endocrine therapy and might be expected to carry a poorer prognosis (Leake *et al.*, 1981*a*).

It would be quite wrong to draw conclusions from experience with breast cancer about the outcome of endocrine therapy or the prognosis in carcinoma of the cervix, simply because some of both types of cancer contain REs. The concentrations of both $RE_C$ and $RE_N$ are lower in carcinoma of cervix than in carcinoma of the breast (Leake *et al.*, 1981*b*; Pegoraro *et al.*, 1980) and other, more subtle, differences may exist. However, it is most encouraging to note that two controlled studies of oestrogen therapy combined with radiotherapy, as the primary treatment of carcinoma of the cervix, have shown a clear and substantial improvement in the 5-year survival of patients given oestrogens (Runge, 1959; Sugimori *et al.*, 1976).

If the mode of action of oestrogens in carcinoma of the cervix is similar to that in carcinoma of breast, anti-oestrogens may be equally effective (Ingle *et al.*, 1981) and the measurement of progesterone receptors may also be valuable (Barnes *et al.*, 1980).

This study was supported by the S.A. Medical Research Council through the Preclinical Diagnostic Chemistry Research Group.

### REFERENCES

ANDERSON, J. N., PECK, E. J. & CLARK, J. H. (1974) Nuclear receptor oestradiol complex: A requirement for uterotrophic responses. *Endocrinology*, **95**, 174.

ANDERSON, J. N., PECK, E. J. & CLARK, J. H. (1975) Oestrogen-induced uterine responses and growth: Relationship to receptor oestrogen binding by uterine nuclei. *Endocrinology*, **96**, 160.

BARNES, D. M., SKINNER, L. G. & RIBEIRO, G. G. (1979) Triple hormone receptor assay: A more accurate predictive tool for the treatment of advanced breast cancer? *Br. J. Cancer*, **40**, 862.

BUSH, I. E. (1952) Methods of paper chromatography of steroids applicable to the study of steroids in mammalian blood and tissues. *Biochem. J.*, **50**, 370.

HÄHNEL, R., MARTIN, J. D., MASTERS, A. M., RATAJEZAK, T. & TWADDLE, E. (1979) Oestrogen receptors and blood hormone levels in cervical carcinoma and other gynaecological tumours. *Gynecol. Oncol.*, **8**, 226.

INGLE, J. N., AHMANN, D. L., GREEN, S. J. & 8 others (1981) Randomized clinical trial of diethylstilboestrol *versus* tamoxifen in post-menopausal women with advanced breast cancer. *N. Engl. J. Med.*, **304**, 16.

KATZENELLENBOGEN, B. S. & LEAKE, R. E. (1974) Distribution of the oestrogen-induced protein and of total protein between endometrial and myometrial fractions of the immature and mature rat uterus. *J. Endocrinol.*, **63**, 439.

LEAKE, R. E., LAING, L., MCARDLE, C. & SMITH, D. C. (1981*a*) Soluble and nuclear oestrogen receptor status in human breast cancer in relation to prognosis. *Br. J. Cancer*, **43**, 67.

LEAKE, R. E., LAING, L., CALMAN, K. C., MACBETH, F. R., CRAWFORD, D. & SMITH, D. C. (1981*b*) Oestrogen-receptor status and endocrine therapy of breast cancer: Response rates and status stability. *Br. J. Cancer*, **43**, 59.

MCGUIRE, W. L., ZAVA, D. T., HORWITZ, K. B., GAROLA, R. E. & CHAMNESS, G. C. (1978) Receptors and breast cancer: Do we know it all? *J. Steroid Biochem.*, **9**, 461.

O'MALLEY, B. W. & MEANS, A. R. (1974) Female steroid hormones and target cell nuclei. *Science*, **183**, 610.

PEGORARO, R. J., SOUTTER, W. P., JOUBERT, S. M., NIRMUL, D. & BRYER, J. V. (1980) Nuclear and cytoplasmic oestrogen receptors in human mammary carcinoma. *S. Afr. Med. J.*, **58**, 807.

RUNGE, H. (1959) Zusätzliche hormonbehandlung des Krebses. *Arch. Gynäkol.*, **193**, 122.

SANBORN, B. M., KUO, H. S. & HELD, B. (1978) Oestrogen and progesterone binding site concentrations in human endometrium and cervix throughout the menstrual cycle and in tissue from women taking oral contraceptives. *J. Steroid Biochem.*, **9**, 951.

SCATCHARD, G. (1949) The attraction of proteins for small molecules and ions. *Ann. N.Y. Acad. Sci.*, **51**, 660.

SOUTTER, W. P., HAMILTON, K. & LEAKE, R. E. (1979) High affinity binding of oestradiol-17$\beta$ in the nuclei of human endometrial cells. *J. Steroid Biochem.*, **10**, 529.

SUGIMORI, M., TAKI, I. & KOGA, K. (1976) Adjuvant hormone therapy to radiation treatment for cervical cancer. *Acta Obstet. Gynaecol. Jpn*, **23**, 77.

SYRJÄLÄ, P., KONTULA, K., JÄNNE, O., KAUPPILA, A. & VIHKO, R. (1978) Steroid receptors in normal and neoplastic human uterine tissue. In *Endometrial Cancer*. Ed. Brush *et al.* London: Baillière Tindall. p. 242.

TERENIUS, L., LINDELL, A. & PERSSON, B. H. (1971) Binding of oestradiol-17$\beta$ to human cancer tissue of the female genital tract. *Cancer Res.*, **31**, 1895.